# The Large-Scale Implementation of a Health Information System in Brazilian University Hospitals: Process and Outcomes

**DOI:** 10.3390/ijerph20216971

**Published:** 2023-10-25

**Authors:** Clarissa Carneiro Mussi, Ricardo Luz, Dioni da Rosa Damázio, Ernani Marques dos Santos, Violeta Sun, Beatriz Silvana da Silveira Porto, Gabriel Oscar Cremona Parma, Luiz Alberto Cordioli, Robert Samuel Birch, José Baltazar Salgueirinho Osório de Andrade Guerra

**Affiliations:** 1 Postgraduate Program in Administration, University of Southern Santa Catarina, Palhoça 88137-270, Brazil; ricardoluzz@yahoo.com.br (R.L.); dionidamazio@gmail.com (D.d.R.D.); gabriel.parma@animaeducacao.com.br (G.O.C.P.); luiz.cordioli@animaeducacao.com.br (L.A.C.); jose.baltazarguerra@animaeducacao.com.br (J.B.S.O.d.A.G.); 2 Postgraduate Center in Administration, Federal University of Bahia, Salvador 40110-903, Brazil; emarques@ufba.br; 3 School of Arts, Sciences, and Humanities, University of São Paulo, São Paulo 03828-000, Brazil; violeta@usp.br; 4 Center for Health Sciences, Federal University of Santa Maria, Santa Maria 97105-900, Brazil; 5 School of Engineering, University of Liverpool, Liverpool L69 3GH, UK; rsb123@liverpool.ac.uk

**Keywords:** public health, hospital management, health information technology, hospital information system, heath information system, development, university hospital, teaching hospital, large-scale implementation

## Abstract

Governments around the globe are paving the way for healthcare services that can have a profound impact on the overall well-being and development of their nations. However, government programs to implement health information technologies on a large-scale are challenging, especially in developing countries. In this article, the process and outcomes of the large-scale implementation of a hospital information system for the management of Brazilian university hospitals are analyzed. Based on a qualitative approach, this research involved 21 hospitals and comprised a documentary search, interviews with 24 hospital managers and two system user focus groups, and a questionnaire of 736 respondents. Generally, we observed that aspects relating to the wider context of system implementation (macro level), the managerial structure, cultural nuances, and political dynamics within each hospital (meso level), as well as the technology, work activities, and individuals themselves (micro level) acted as facilitators and/or obstacles to the implementation process. The dynamics and complex interactions established between these aspects had repercussions on the process, including the extended time necessary to implement the national program and the somewhat mixed outcomes obtained by hospitals in the national network. Mostly positive, these outcomes were linked to the eight emerging dimensions of practices and work processes; planning, control, and decision making; transparency and accountability; optimization in the use of resources; productivity of professionals; patient information security; safety and quality of care; and improvement in teaching and research. We argued here that to maximize the potential of information technology in healthcare on a large-scale, an integrative and cooperative vision is required, along with a high capacity for change management, considering the different regional, local, and institutional contexts.

## 1. Introduction

The performance of hospital institutions and the quality of patient care have been maximized due to the implementation of health information systems (HIS) based on information and communication technology (ICT). Among these systems, the hospital information systems facilitate hospital management by increasing efficiency, quality and availability of services provided to the patient while improving the use of healthcare resources [1,2,3].

Initiatives to implement HIS are often permeated with challenges, especially when it comes to government implementation programs in national networks comprised of different health institutions [4,5]. Given their large-scale scope, programs of this nature are commonly characterized as complex or mega-projects making it necessary to understand and manage the multiple stakeholders and organizations involved, often with different interests and perspectives [4,6].

Many countries have undertaken initiatives to implement large-scale health information systems to improve the quality of health services [7]. Although these countries have invested significant financial resources when implementing health information technology nationally, there are frequent reports of difficulties or barriers involving technical, human, social, organizational, and environmental issues [4,7,8].

Since the 1970s, the Brazilian government has been developing HIS with the objective of computerizing its data and obtaining reliable information to support the management and planning processes of the Unified Health System (SUS). The SUS is currently one of the largest and most complex public health systems in the world, providing comprehensive, universal, and free access to healthcare. The network that makes up the SUS is broad and covers primary, medium, and high-complexity care, urgent and emergency services, hospital care, epidemiological, sanitary, and environmental surveillance actions and services, and pharmaceutical assistance. The SUS comprises several hospital institutions, including the network of university hospitals.

In line with its purpose of ensuring improvements in public health, SUS established, in 2010, the National Program for the Restructuring of Federal University Hospitals (REHUF—Programa Nacional de Reestruturação dos Hospitais Universitários Federais). The objective of REHUF was to “create material and institutional conditions so that the federal university hospitals can fully perform their functions regarding the dimensions of teaching, research and extension and healthcare” [9]. Brazil has 50 federal university hospitals integrated into the SUS and linked to 35 Federal Institutions of Higher Education [10]. Brazilian university hospitals (HU—Hospital Universitário) play a fundamental role in the country’s healthcare system, providing medical assistance, conducting scientific research, and promoting the education of healthcare professionals. These hospitals are considered centers for training human resources, and developing technology for healthcare and are heterogeneous in terms of their capacity, technological resources, and scope of care [9].

With the establishment of REHUF, Brazilian university hospitals that were managed formerly by the universities to which they were linked, are now under the control of the Brazilian Company of Hospital Services (EBSERH), which was created by the federal government to manage this network of public university hospitals. As part of the centralized management actions, there is the national implementation of a computerized hospital information system called “Management Application for University Hospitals” (AGHU —Aplicativo de Gestão para Hospitais Universitários). The system aims to standardize the care and administrative practices of the university hospitals in the federal network and to allow the creation of national indicators, facilitating the development of common improvement programs for all these hospitals [9].

Despite the efforts made over several years by the national program to implement the AGHU in university hospitals, the process is still in progress [10]. In this context, we sought to answer the research question: how do managers and users perceive the process and outcomes of the large-scale implementation of a hospital information system for the management of federal university hospitals? More specifically, the intervening aspects in the process of implementing the AGHU (facilitators and limiters) and the outcomes obtained from the use of this system by hospitals, from the perspective of the managers and health professionals involved are analyzed.

Although there is ample literature on the implementation of HIS based on ICT, these investigations are generally limited to just one or a few sites while investigations of government initiatives on a national scale are still limited [4,11,12]. Furthermore, these studies focus mostly on initiatives in developed countries with only a few exploring national programs in developing countries, as well as in university hospitals [7].

The challenges in these programs of implementation become even more complex in developing countries. Despite the growing effort dedicated to such initiatives, there is still limited evidence, demonstrating the contribution of these health information systems to improving health outcomes and enhancing the well-being of economically disadvantaged individuals in these nations [13]. Therefore, it remains necessary to maximize knowledge and understanding of the perspectives in this process [14].

This paper aims to fill a recognized knowledge gap within this complex context in a Latin American country of continental dimensions. Although the Brazilian government has been undertaking several actions to support the implementation of ICT in health that have contributed to economic, social, and human development [15], little is known about the process and outcomes of the national implementation of AGHU in Brazilian university hospitals.

We believe that this study can contribute to the field of the large-scale management, implementation, and evaluation of HIS in developing countries, bringing insights that may assist in the success of implementing similar systems in other contexts. In addition to Brazil’s level of development, characteristics such as its vast territory and the complexity of university hospitals that not only focused on care but also on teaching and research, shed further light on the theoretical advances on the subject and the challenges in this governmental program.

The study was conducted across 21 university hospitals. We gathered data through in-depth interviews with key managers from five hospitals as well as through focus groups involving key users. Additionally, we administered questionnaires to both managers and users of the 21 participating hospitals in the research. Both qualitative analysis and descriptive statistics were employed as complementary approaches. As a result of this analysis, a set of intervening aspects influencing the system’s implementation process emerged. These aspects were examined at three distinct levels: macro, meso, and micro. Furthermore, the findings pertaining to system usage in hospitals were elucidated based on the emerging themes identified in the analysis.

## 2. Large-Scale Implementation of Health Information Systems

HIS can be defined as a set of interrelated components that collect, process, store, and distribute information to support the decision-making process and control of health organizations, at their strategic, tactical, and operational level [16]. Hospital information systems, which are considered a specific type of HIS [17], integrate hospital administrative and care processes through ICT with the aim of maximizing operational efficiency and the quality of care [16,18].

It has been the belief of many governments that the key to modernizing their health systems is to invest in HIS [19]. The adoption of HIS has been perceived globally as a way to increase efficiency in the management of public health policies and institutions. More specifically, through these initiatives, nations expect improvements in the efficiency and safety of patient care; privacy of health information; greater potential for patient choice about their care; improvements in citizens’ access to health services; integration and sharing of information between different organizations and health professionals; advances in the definition of indicators and public health policies; cost reduction; more consistent and efficient use of health resources; and reduction of the gap between the health care demand and supply [7].

Although there are reports of success from the implementation of these large-scale government HIS programs [20,21] alongside the high investment in supportive technologies, there are successive and frequent cases of difficulties. Deficiencies in the planning process, failure to consider regional aspects in the design of systems and in their implementation of processes, and the centralization of decisions are considered critical factors [22,23,24]. When analyzing the difficulties faced by 24 countries, 34 main difficulties were identified [7] that related mostly to the ability to manage programs, both at the governmental and organizational levels, as well as reconciling the different needs of the various stakeholders. In more than half of the countries examined, the barriers that appear are related to a lack of standardization and interoperability (leading to difficulties in integrating systems), regional and supplier factors; resistance of health professionals and users; and project financing/budgetary constraints.

There is a growing understanding that the introduction of technology on a large scale into complex organizational systems, such as hospital institutions, is not a straightforward linear process. Rather, it is dynamic in nature, often involving multiple cycles of interaction, as technological, social, and organizational dimensions that align or misalign gradually over time [12,25]. Undertakings of this nature involve more than just replicating a technological system among different institutions but include the definition, by governments, of policies and standards that encourage the convergence of public and private interests in the development of an effectively functional national system [26].

The process of implementing HIS, in order to achieve the objectives proposed by the use of the system, should be conducted in such a way as to maximize potential gains and mitigate losses [27]. The projection of risks and evaluation of the variables involved in the process can avoid the generation of unforeseen events and unwanted results. In this way, the quality of the implementation process has as significant an impact as the quality of the system itself [28].

## 3. Research Framework

**Figure 1:**
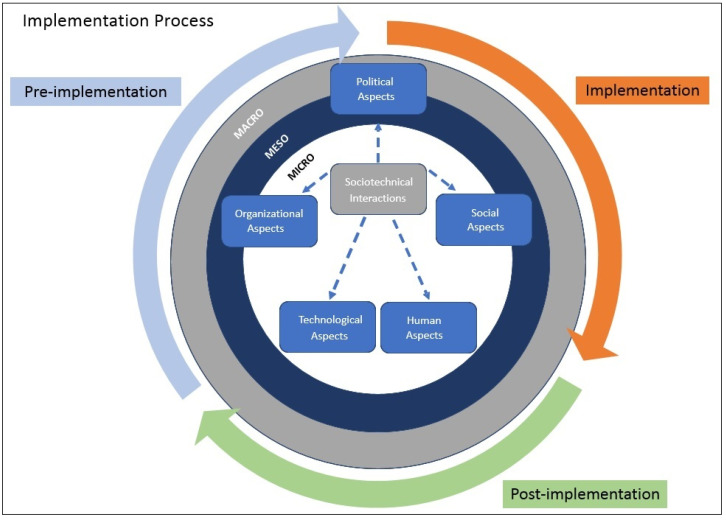
Research Framework. Source: Adapted from [32].

The introduction of health ICT on a large scale is affected by a range of aspects at the macro, meso, and micro levels [29,30]. These three levels contain aspects of a socio-technical (technological, organizational, social, political, and human), interdependent, and interacting nature, which format, enable, and restrict the introduction of HIS [16,24,25] in different stages of its development cycle comprising pre-implementation, implementation, and post-implementation [12,31]. Figure 1, which represents the theoretical research framework, illustrates this dynamic.

The evaluation of the implementation process of an HIS should not be limited to the evaluation of aspects directly resulting from the continuous use of the technology in the application, but, more broadly, it requires an information structure, that also considers the various phases of the implementation process [12,31]. Thus, for the purpose of this research, we considered the temporal aspect [25,31], where the implementation process is represented by the pre-implementation, implementation, and post-implementation phases of the system [31,32].

The pre-implementation phase corresponds to the period prior to the start of the actual implementation of the system. It is characterized by the organization’s preparation for implementation. The implementation phase corresponds to the period of effective implementation of the system. The post-implementation phase, on the other hand, corresponds to the period of use of the system. This process is circular, as the implementation of new features of a system can restart the process.

In addition, it is recognized that the multiple and complex interactions between socio-technical aspects result in potential influences on the process of system implementation [12,17,24,25,28,32]. These influences can be understood at three levels of analysis: macro, meso, and micro. The first level (macro) brings together the aspects of the broader context of the system’s implementation. It is related to the social, economic, political, and competitive environment in which change occurs [33], involving, for example, national and regional priorities and policies [29,30]. The second level (meso) refers to the structural, cultural, and political context within the organization in which the introduction of change takes place [33]. Although programs for the introduction of large-scale health information systems are often the result of national initiatives, these systems are implemented in specific regions, organizations, and environments with peculiar contextual characteristics. Thus, the meso level involves, for example, an organizational background where the technology is being implemented, work processes, and routines [29,30]. The third level (micro) comprises the aspects inherent to the individuals who make use of the technology and who are affected by it, as well as the attributes of the technological tool itself. In this way, this level brings together issues such as material properties of technology, individual attitudes and concerns, and interpersonal influence [29,30].

Therefore, the research presented here aims to understand the government program for the implementation of the AGHU in university hospitals in Brazil, based on the analysis of the aspects that affected the implementation process, at the macro, meso, and micro levels, and verify its outcomes, using the methodology described in the next section.

## 4. Materials and Methods

**Figure 2:**
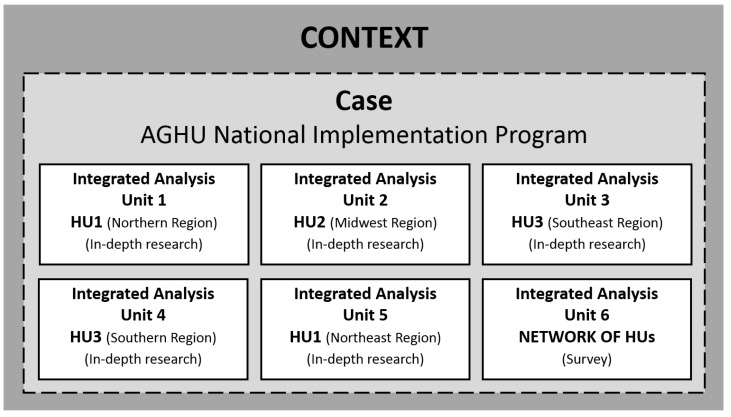
Case study design.

The research was based on a qualitative approach [34] and a case study strategy [35,36]. The investigation was designed as a single integrated case, that is, it involved multiple units of analysis [36], as shown in Figure 2.

The following inclusion criteria were defined for the participation of the hospitals in the research: (1) being affiliated with the EBSERH network; (2) making use of the AGHU or part of it and (3) accepting to participate in the research by expressing their intention by meeting the ethical requirements provided. Of the 40 hospitals in the network, 3 did not meet the second criterion at the time of the study, and 16 did not meet the third criterion, totaling a set of 21 participating hospitals.

The analytical units 1 to 5 (Figure 2) were defined with the objective of obtaining an in-depth understanding of the opinions of the subjects regarding the process and the outcomes of the implementation of the AGHU. For the selection of these 5 hospitals, the following criteria were defined: (a) one hospital from each region of the country, in order to incorporate into the research the regional and territorial differences where the HUs are inserted, (b) the hospital with the largest number of AGHU modules in the region of the country where it is located, in order to consider an in-depth analysis of the most advanced hospitals in the implementation and use of the system in their region. Table 1 presents some characteristics of these hospitals.

Additionally, a sixth analytical unit (Figure 2) was included in order to provide an overview, diagnostic, and exploratory within a wider network of 21 HUs in Brazil. Table 2 presents some consolidated data from these 21 hospitals.

Data collection was carried out through interviews, questionnaires, and focus groups as well as publicly available documentary sources on government and HU sites, manuals, and pre-implementation and implementation reports.

Semi-structured interviews were used to obtain an in-depth understanding of the subjects’ perceptions in the context of the 5 university hospitals. 24 interviews were carried out with the main managers of each of the hospitals: superintendent, healthcare manager, teaching and research manager, administrative manager, and head of the research and technological innovation management sector. The average duration of the interviews was 1 h and 15 min, resulting in a total of 29 h and 40 min of recordings and 867 pages transcribed for analysis. The script for each interview was structured in five topics (Table 3) based on the theoretical framework shown in Figure 1.

Additionally, in two of these HUs, focus groups were held [37] with AGHU users from different sectors who used it frequently in their work activities, comprising a total of 10 users with one of the authors of this research acting as a moderator. These two focus group sessions totaled 1h18min of recording time.

In the analysis of unit 6 (with 21 HUs), questionnaires were used in the survey with the purpose of obtaining a general and exploratory view of the opinion of managers and users regarding the process of implementing AGHU and its outcomes in HUs distributed nationally. The survey was conducted using SurveyMonkey software and with the questionnaire being submitted to care providers and administrative staff of the hospitals. A total of 736 responses, connected with the 21 participating HUs, were received. Of the total respondents, 163 work in management positions, and 573 are AGHU users who do not perform management activities. 570 respondents belong to the care staff (doctors, nurses, pharmacists, nutritionists, and psychologists, among others) of which 74 are residents. Others work in the administrative area (134) and IT (32).

The questionnaire consisted of open and closed questions structured in four sections: (i) characterization of respondents, (ii) national AGHU program—aspects of the broader context of system implementation (macro level), (iii) planning, implementation, and use of the AGHU—aspects inherent to the structural and political context of the hospital (meso level) in which the system was implemented, and aspects inherent to individuals who make use of IT in health and/or who are affected by it, to the technology itself and to the tasks performed (micro level), (iv) outcomes—outcomes obtained using the AGHU.

The open questions allowed the respondent to express, in their own words, the advantages, disadvantages, and difficulties resulting from the use of the system. The closed questions in the questionnaire were based on references [7,29,30,32] and addressed the aspects presented in Table 4.

The data collection took place in a dynamic interaction with its analysis, which is common practice for research with a qualitative approach, and considered two main dimensions: (i) intervening aspects in the implementation process and (ii) process outcomes. An inductive logic was followed, through coding and categorization procedures [38,39] to analyze data from interviews, focus groups, questionnaires (open questions), and documents. In the case of the dimension referring to the intervening aspects, the three levels of macro, meso, and microanalysis and phases of the implementation process were considered, namely pre-implementation, implementation, and post-implementation [12,31]. A qualitative data analysis was performed using the NVivo software, version 12 (developed by QSR International, Burlington, MA, USA). Additionally, descriptive statistics were used to analyze the questionnaire data in order to complement and validate the findings from the qualitative analysis. The methodological or methodological triangulation [40] provided the linkage of evidence from multiple sources, namely interviews, focus groups, questionnaires, and documentary research.

**Figure 3:**
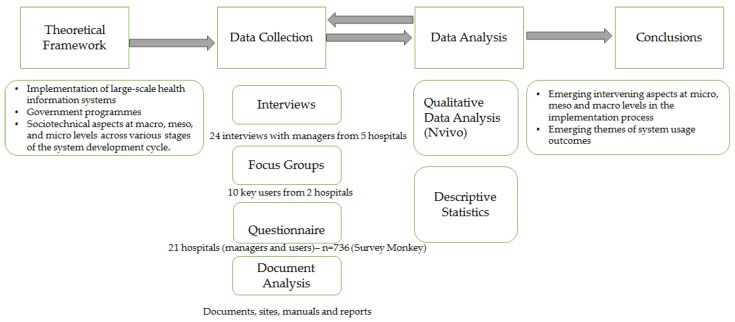
Stages of the research.

Figure 3 presents the stages of the research.

The research was conducted in accordance with Resolution CNS 466/12 [41], which provides guidelines and regulatory standards for ethics in research involving human beings in Brazil. The research was approved by the research ethics committee of the proposing university and by the ethics committees of the university hospitals involved. To maintain anonymity, code M was used in the next sections to refer to the managers and code U to refer to users who participated in the research.

## 5. Results

The AGHU is an integrated hospital information system built using a modular design, covering care processes, administrative processes, operational controls, workflows, and analysis of hospital information and indicators [10]. These modules include patients and online medical records; hospitalization; multidisciplinary evolution; drugstore; stock; examinations; patient control; nursing prescription; doctor’s prescription; administrative outpatient clinic, care outpatient clinic; digital certification; surgeries; infection control; nutrition; purchasing; and billing [10].

The AGHU implementation process was found to be affected by a set of intervening aspects that acted as facilitators and/or limiting aspects at different levels of analysis. Eight themes relating to aspects of the broader context emerged as those that affected the implementation of the system (macro analysis level), as shown in Table 5.

The national public policy for the restructuring of the HUs in Brazil and the institution of EBSERH as the centralized management body of these hospitals led to their organization into a network and the consolidation of a single management and IT policy. This, in turn, prompted the implementation of a standardized system as the AGHU. However, the policy of centralizing the management of HUs was initially a reason for conflict arising from various groups and professional interests, thus the relationships between stakeholders influenced the implementation of the AGHU: “*adherence to EBSERH generated conflicts in varying proportions in each of these hospitals. These conflicts ended up contaminating institutional motivation and often compromised the implementation of such a program*” (G1). Thus, the prerogative of voluntary adherence by HUs in Brazil towards a centralized management made joint planning with all of them for the implementation of AGHU unfeasible. This contributed to delays and differences between the number of modules implemented in each of the hospitals in the network over the course of some years. Furthermore, there were divergent opinions among the managers themselves regarding the adoption of a standard system for all Brazilian HUs. In addition to targeted policies, some managers mention as a limiting factor the lack of definition of a single e-health policy for the entire SUS network and interoperability standards between systems that would enable the adoption of a single electronic medical record system throughout the country. “*Speaking of the larger government plan, of AGHU’s integration with the other systems, this one I haven’t seen yet. So, for me it’s very difficult. It is extremely complicated to make the database talk to the official systems of the Ministry of Health, not to mention the municipal systems. One thing doesn’t talk to the other.*” (G8).

Another challenge relates to the vast territorial extension of Brazil and the differences at regional and hospital levels where the system was implemented. The contextual differences of each hospital in terms of maturity of previous systems, size, physical structure, organizational culture, care processes, clinical protocols, and even languages were mentioned as limiting the process: “*you have 40 hospitals with different epidemiological profiles, with different cultures, with different ways of working, each one is very different, the regions are different, it is difficult, it is not like a bank where the processes are the same in all places.*” (G9). These differences demanded the need to resolve compatibility issues for the adoption of a standardized system, implying a longer development and implementation time.

The national policy for restructuring the HUs provided funding to increase the human and material resources of each one in the network, contributing to the construction of an environment and infrastructure conducive to the introduction of the AGHU: “*The investments that the hospitals received were very conditioned to the issue to provide this foundation for AGHU to work well.*” (G20). In addition, the availability of a budget dedicated especially to information technologies has been contributing to the implementation and use of the system, although it is clear that financial resources are still not considered sufficient for all the demands of some HUs.

The government support and involvement arising from policies and actions led by EBSERH, materialized by “*investments*”, “*organizational structuring*”, “*support in team composition*”, “*administrative and logistical support*”, were seen as a facilitator. However, government support was influenced by instability in the country’s political and economic scenario. Presidential changes, including an impeachment process, and the change of managers provoked reorientations and even threats to the continuity of the program. This scenario generated insecurity and contributed to delays in the development and implementation of the system in the country’s hospitals: “*With each change, with each change of managers, there were breaks in the regularity of implementation, ups and downs. We even had administrations that started to analyze AGHU, and in a way they proposed the acquisition of other applications*” (G15). A return to stability in the management structure of the HUs and government commitment to the implementation of the AGHU was perceived by the government that started in 2019: “*So we did feel that the forces took a different course, to not have this insecurity, so there is more perennial planning.*” (G22).

Different aspects related to the governance of the implementation process that influenced the implementation of the AGHU system emerged in the research. The adoption of a top-down approach [28] and the institution of the AGHU as mandatory, for example, limited the participation of the HUs in defining the system to be adopted. On the other hand, it was seen as favorable, especially by the HUs that did not use computerized systems or that had incipient systems, since it accelerated the local decision-making process regarding the choice of system to adopt. However, the limited autonomy given to the local IT teams is perceived as a difficult aspect, especially when considering the small team at the headquarters as well as limitations regarding this team’s knowledge of the care processes. Such deficiencies affected the speed of development, implementation and resolution of technical problems of the AGHU and resulted in the creation of barriers and resistance: “*delays in improvements end up wearing people’s patience (G24). Because we have a demand generation system, and why do I say that it doesn’t work: because they have a small team to deal with 40 hospitals.*” (G19). The communication process between the managing body and the HUs in the network is understood by some to be effective, especially during the implementation of the modules. However, others perceive that it is limited and needs improvement in terms of agility in generating responses to the demands of the HUs and communicating a comprehensive plan, considering the development and introduction of new modules and the reality of each hospital: “*I think the communication is flawed. There is certainly a breakdown in communication. At the very least, if you ask for an improvement, you should know: we’re going to work on it or we’re not*” (G11). These aspects affected local planning and preparation and created expectations beyond those that potentially could be met by the system. Likewise, although there are some initiatives to promote the sharing of knowledge and lessons learned among the HUs, and these have been contributing favorably to the implementation, reports about the need for a clear policy instituted for the sharing of experiences are identified. The training of the HUs to use the system provided by the headquarters is seen favorably during the implementation of new system modules: “*The period they spent here in training allowed people to have a better view of the system and motivate themselves to use it more assertively* (G22)”. However, a continuing training program from the management body, in the opinion of some participants, could improve the use of the system and contribute to minimizing resistance. Another aspect, concerning the evaluation of the program’s performance by the managing body, considers audits of a more quantitative nature being carried out at the HUs, and a continuous performance evaluation program is seen as being necessary regarding the process of implementing the AGHU. This includes considering the regular analysis of costs, risks, and benefits, as well as the evaluation of needs and satisfaction of users.

One of the most frequently mentioned factors that hindered the implementation and use of the system is related to the design and implementation plan of the system, more specifically the implementation of an incomplete system (with all modules and functionalities available). This fact led to the adoption of local solutions to fill the system’s functional gaps and created frustrations for users and managers, due to expectations not being met by the system. The effects of the non-delivery of a complete solution were aggravated by the delay in the process of developing and implementing new modules: “*the difficulty of getting responses, or this non-response, facilitated everything I said: creating tools, creating other resources, developing other programs that talk to AGHU, because the response did not happen, or it came very late*” (G3). Limitations regarding the documentation of the system, the implementation process and lessons learned were identified as generating difficulties: “*the great disadvantage of AGHU today is its documentation, which is very incipient, it is very poor. We do not have a documentation process, there were a number of things that the hospital went through to implement the exam module, no one in the EBSERH network knew how to answer that problem and there was no documentation either, so we were left with trial and error for several weeks until we were able to implement all of this.*” (G9). Limitations related to a contingency plan in case of system unavailability are also of concern. Planning and complying with module implementation schedules is seen as a facilitator by some who understand that the schedules are feasible, and defined together with the affected areas while considering the available resources. However, this perception does not apply to all hospitals. Some still question the development and implementation time of the system: “*The development and implementation time, that’s all I look at. I was promised the software for 2017, in 2018 I didn’t have it, in 2019, at the end of the year, I managed to run it. And I got only a part, missing a part. So that’s it, that’s the difficulty of following a schedule*” (G6).

The process of implementing the AGHU on a national scale arrived at HUs at different times, in terms of established cultures and the level of development of their organizational structures. Nine themes emerged related to aspects of the organizational context of the HUs that affected the implementation of the system (meso analysis level), as shown in Table 6.

The support and commitment of the hospital’s top management to the implementation process and its objectives, the firmness of purpose, and the commitment to monitoring the process and addressing demands for improvements, as well as the alignment between managers, facilitated the implementation of AGHU better in some hospitals rather than in others. This contributed to the commitment and engagement of users; as one user expressed it: “*But, one facility that we had, is that the superintendent gave a great support to it, so the whole management collaborated a lot for the process to become effective*” (U1). But, the same was not observed in some hospitals where it became a hindrance, as seen in the following quote: “*lack of commitment of the managers of the affected areas*” (U37), “*lack of interest of the managers in the initial implementation of the system*” (U9), “*lack of governance participation in the digital transition process, generating excessive responsibilities for IT*” (G13).

A receptive and even desired change environment, especially in the HUs that did not use computerized management systems or with inefficient systems, contributed to the national initiative to implement AGHU. Furthermore, management control transfer to EBSERH characterized the beginning of a transition in the cultures of the different hospitals, especially due to the arrival of new professionals which contributed to the implementation of the system. However, the need for changes in institutionalized work practices generated resistance and hindered the implementation and adoption of the system: “*a culture of not reading the evolution of colleagues from another area nor reading the consultancy response*” (U48); “*a culture of how things were done, the way of working and having to adapt to a completely computerized technology, to evolve to an electronic process, I think that is the greatest difficulty*” (G15).

The normalization of the local HUs’ hierarchical structures contributed to the implementation of a standardized system such as the AGHU. In this structure, the local IT area occupies a position directly linked to the HU top management, which also facilitated the process of implementing the AGHU: “*IT is a sector, but it is directly subordinated to the superintendence. This contributes significantly*” (G22). However, it must be considered that, in some HUs, the IT area does not participate in local collegiate groups, which, according to the research participants, could contribute to a greater alignment of the area with the other areas of the HU. Still, in some HUs, it was identified that limitations in the local definition of flows and work routines compromised the agility of the activities in the AGHU.

People management difficulties also emerged in the records of research participants. The different rules for hiring professionals, resulting in part from EBSERH’s centralized management, constituted a hindrance, being yet another issue to manage in the implementation of the AGHU. Moreover, staff turnover, characteristic of the context of university hospitals, is also composed of a wide range of resident professionals who work temporarily in the hospital. On the other hand, a large number of employees at retirement age and the consequent entry of new professionals was considered a facilitator given the differences between these professional profiles: “*another thing that facilitated it was that… people were retiring. So those who created the ‘I don’t know, I don’t want to’ barrier have retired. A new one comes in, when a new one comes in… he has the perception of ‘I have to use the system, this is the official hospital system* ” (G8). Even with a contingent of new admissions, work overload is mentioned in some HUs as compromising the implementation and use of the AGHU: “*The great difficulty we have is the work overload and the reduced team. So people have to work hard and obviously this creates stress, some are at their limits*” (G7). Also, problems in defining system user profiles due to the lack of a clear definition of professional attributions interfered with the execution of daily activities at some HUs.

In some HUs, local initiatives for the change management process played a facilitating role, enhancing user engagement and minimizing resistance. Among these initiatives, were: communication and awareness programs; actions aimed at understanding difficulties, identification and individualized treatment of resistance; wide dissemination of positive results obtained with the use of AGHU; groups of young professionals, residents, and experienced users as multipliers and encouragers of technology use; adoption of integration practices for new professionals; ongoing training programs; key users of the areas in the implementation teams, and prioritization and institutionalization of the use of the system by the HU top management: “*some groups offered a little more resistance, but we managed to win over some within the group by that thing of sharing information, transparency of information, acknowledging the difficulties of the system, listening to the guy about the reason for the resistance, so all this helped*” (G21). In other HUs, limitations in initiatives to change management hampered the implementation process. Thus, a user expresses himself: “*We arrive ‘look, nice to meet you, I’m AGHU and turn around*”. (U8)

The creation of local management committees for the implementation process (local governance of the implementation process), composed of professionals from different areas as well as IT professionals, contributed to the implementation of AGHU. In some HUs, part of the success of the AGHU implementation process is credited to the representativeness and effective participation of the various sectors in the management committee. Thus, the participants expressed their opinion about the committee: “*there are many more users than IT professionals. All facets of assistance were seen exactly and placed on the committee, and this has facilitated the implementation process a lot*” (G11). Although this was EBSERH’s general guideline, in hospitals where the performance and level of action of these committees were not effective, governance responsibilities fell under the local IT sector, which constituted a difficulty: “*became very IT-focused, as if the system were IT’s responsibility.*” (G9). It was identified as a facilitating aspect, in some HUs, the effectiveness of communication with the user, as well as their participation in the implementation and addressing of improvement needs: “*It had results, it was a two-way street for the solution of the problems detected by the teams, and that motivated the teams*” (G1). The same did not occur in other HUs, which showed difficulties in communication with users. Local initiatives by the HU to share experiences with their peers, especially the more advanced HUs in the process of implementing the AGHU, favored inter-organizational collaboration, facilitating this process: “*If my team is experienced in a certain module, and the other team is having difficulties in this module, allow the experienced team to help the other team. We have already done this…*” (G1).

Other aspects that influenced the implementation of the system are related to the IT resources and capabilities of each hospital. The limited physical IT structure of most HUs was characterized as a hindrance, demanding funding, updates, and, consequently, longer implementation time. This fact was further aggravated by the old physical infrastructure of some HU facilities, whereas the fact that many HUs with outdated rudimentary systems with poor functionality contributed to an environment conducive to their replacement and made them more receptive to the implementation of AGHU. The same did not occur in HUs that used effective systems, mostly developed internally and customized to hospital processes and routines: “*There were some systems that were developed by the university’s own IT that served some areas very well. When AGHU arrived, it did not show the same results, the same facilities*” (G19). The reduced number of members of the local IT teams, as well as the limited expertise in healthcare, are pointed out as aspects that made it difficult both to implement the system and to provide technical support: “*Look, today IT is sacrificed a lot, it has a very big responsibility, and the team is very small*” (G7). “*I think the IT team needs to mature a little in terms of this knowledge*” (G24). In some hospitals, aspects such as the availability of permanent technical support, agility, and commitment to the implementation of AGHU by the local IT team contributed to the adoption and use of the system. “*today we have an IT team that is very active, very participatory in our process, which they really fight for the implementation of AGHU, they buy the idea.*” (U6). However, in other HUs, technical support has not been effective, as this report illustrates: “*weak and inefficient technical support during implementation and, mainly, after implementation*” (U63).

In some HUs, localized continuing education and training programs for new professionals contributed to the use of AGHU and staff engagement: “*It’s permanent education*” (G3), says a manager. The offer of different training modalities and strategies, such as customized and on-demand training, online training, hands-on, videos and tutorials, establishment of differentiated schedules are mentioned as facilitators of the implementation process. This contributed to the adhesion of a greater number of professionals given the high demand and the dynamics of the care services provided in hospital institutions. In addition, the use of “multipliers” (staff experienced in the use of AGHU) in training activities was an aspect that contributed to the engagement of users and the dissemination of the use of AGHU: “*we created an area, a room called AGHU permanent training. There are people who don’t like that, they like a more personalized treatment, especially the medical staff. That’s why there was the role of the doctor [multiplier], who would go there and sit next to the person, sometimes in the same room where he was working, in his sector, and there he would do personalized training, at the person’s side, which also gave a lot of results.*” (G13). On the contrary, in other HUs, deficiencies in the training processes or even the absence of it acted as obstacles to the use of the system: “*Perhaps, what may have made it more difficult is a question of deeper and more continuous training of the users of the system. I believe that continued training would have facilitated this implementation*” (G19). Local evaluation initiatives, such as monitoring the implementation and use of the AGHU, carried out by some hospitals, provided the identification of areas or situations that compromised the implementation progress, usage statistics, and identification of suggestions for improvements, and others. This early identification made it possible to plan and execute corrective actions, such as awareness events, training, technical support, and reports on necessary improvements, with the direct involvement of the immediate heads of the affected sectors: “*when we identify a place that has a lot of non-compliance with the use of the application, we send the trainers to that area*” (G3). “*We have statistics, but it is our own initiative*” (G11). In addition, the master plan of some hospitals includes indicators related to the AGHU implementation process, many of which are used to report on audits carried out by the national management body.

In addition to the intervening aspects in the implementation of the AGHU at the macro and meso levels, three major themes emerged related to those aspects that affected the implementation of the system at the micro level: technology, individuals and health activities/processes (see Table 7).

Limitations regarding the alignment of technology functionalities with work practices in some HUs and sectors and functional gaps resulting from the implementation of a system without all the developed modules generated resistance and demanded the creation of parallel systems and controls by the HUs themselves: “*for the neonatal ICU, which has a very different prescription process from the medical clinic, which are extremely different patients, the AGHU, within the prescription, it comes with the presentation and then the administration. So, the neonatal ICU staff always complained a lot about the AGHU prescription format*” (G9). In the perception of some participants, the lack of maturity of the system, resulting from a partial or incomplete implementation, has implications for the reliability of users: “*Within a system where I have different things not implemented, modules to be implemented and improved, even I get reliability, I think it still takes a little time and maturity*” (G8). The usability of the system is another aspect brought up by the participants, although in a controversial way in different hospitals and even by different individuals from the same hospital as their quotations illustrate “*simple to work with*” (U9), “*intuitive*” (U1), “*friendly interface*” (U24), “*a language that approaches the language of the health professional*” (G22), “*not very intuitive, it has several tabs, the paths they are not so easy, they are not so direct”* (G12); “*They didn’t think about the practicality of those who are going to use it*” (G8)., technical issues are also mentioned, in some hospitals, as unfavorable to the activities: “*it hangs a lot, losing the progress already made*” (U76), “*slow platform, hinders the progress of the urgency/emergency sector.*” (U65.)

Expectations, experiences, resistance, skills, and motivation of individuals emerged as personal aspects that influenced the implementation of AGHU. On the one hand, skepticism, and unrealistic expectations frustrated by the delivery of a system in stages and the slowdown in its development, generated resistance and made the process difficult. On the other hand, positive expectations such as management qualification and process improvement contributed to the adoption of the system: “*The care staff saw that it was no longer possible for us to keep the physical records the way they are, so that comes greatly facilitating this team’s attitude towards joining the AGHU, to improve processes*” (G24). In addition, previous experiences of users with other similar systems positively or negatively affected their motivation to use AGHU, since comparisons were usually made between both: “*Comparisons with other applications from other hospitals, mainly physicians, do this: I cannot. Why is it like this here? Because you don’t get one at another hospital, which is easier*”. (U1). In the same way, positive or negative experiences with the use of AGHU affect the user’s motivation. Resistance to change by numerous factors was observed as compromising the engagement and adoption of the system. Resistance arising from the change from paper to technology was identified as the perception of lack of alignment between technology and some work practices; the change of systems in use considered effective by a new system; the perception of increased workload and complexity in carrying out tasks using the new system; the demand for relearning to adapt to new tools and work routines: “*For us to get the paper out of some places, is very difficult, until today there are still some groups of resistance, there were some professionals who resisted registering, did not want to use it and stuck to the sheet of paper.*” (G2). In addition, the lack of ability to use computers by some professionals, especially those who are older or have a longer career, has compromised adoption.: “*What I see in the hospital is the challenge for us to make the eldest adapt to the computer.*” (G8). From another perspective, the group of medical and multidisciplinary residents, composed of young professionals in training, showed a natural interest in using and discovering new uses for the technology introduced, acting as technology promoters with other groups: “*There is a facilitator at the university hospital who is the student—the resident—because he is young, because he likes technology, he is not afraid.*” (G4). Interest and personal motivation, or lack thereof, also appear in the statements: “*And the other thing worth saying is the following: we get tired of doing training. Yes, it’s one thing to get tired of doing it, the other thing is adherence to training, isn’t it?*” (G15). It was also identified that the use of the system in a different way from what was intended acted as a difficult aspect. Records not made timely or not elaborated completely, indiscriminate use of “*copy and paste*” are some examples: “*They started copy-pasting. When you start picking up and seeing a lot of medical records, there are things that are very repetitive. The guy saw the system as an advantage for not using it correctly*” (G8). “*Because many times we go to look for information and it was not registered in a timely way. So, this is no longer a system problem, but a user problem; of the informant; of the person who should have recorded that information.*” (G19).

Also at a micro level, the high complexity of hospital activities and processes and the peculiarities of each of the institutions represented an obstacle to implementation: “*it is difficult, it is not like a bank where processes are the same in all locations. At the bank it is the same in the 5 thousand municipalities of the country, the hospital is not, you know?*” (G9). Added to this is the fact that they are university hospitals, thus incorporating academic processes into those of care and administration: “*It is a teaching hospital, so you know that it has all that consultation protocol and such, so this ends up being a hindrance, because we cannot change this reality and we still need to do everything in the system*” (G15).

The intervening aspects identified in the national program affected the outcomes of the program. Although the most advanced hospitals in the implementation of AGHU have obtained several benefits with the use of the system, they were not equally evident in all HUs. Table 8 presents the 8 categories emerging from the collected data that bring together the outcomes perceived after the use of AGHU.

## 6. Discussion

**Figure 4:**
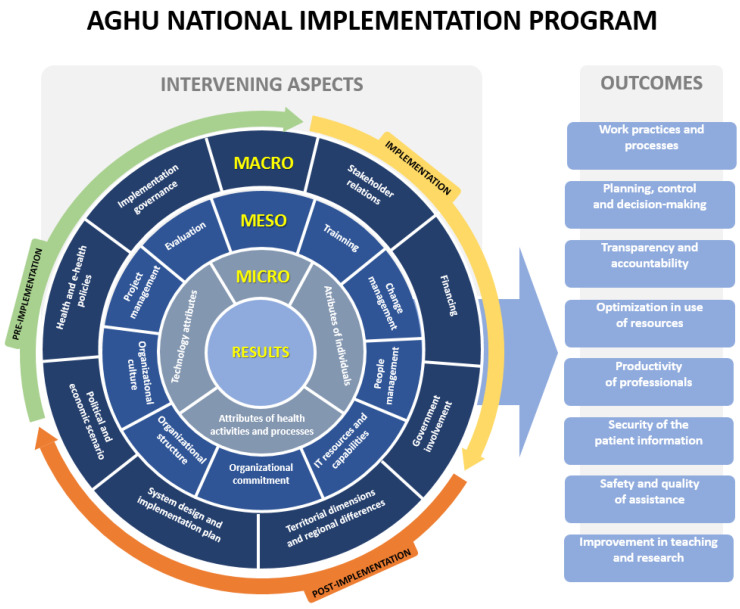
Intervening aspects and outcomes of the national program to implement AGHU.

The research results show that the national program for the implementation of the AGHU in university hospitals was influenced by aspects at the macro, meso, and micro levels that acted both in facilitating and hindering the process and the achievement of the desired results. Figure 4 illustrates a consolidation of these aspects and outcomes that emerged from the research.

As highlighted in the previous literature, national programs for implementing health IT are considered challenging internationally with numerous aspects of different natures acting in this process [4,8,42,43]. More specifically, the implementation of HIS in developing countries depends on the insertion of technologies in the social context of different organizational scenarios [44]. Despite the efforts of more than 10 years since the beginning of the Brazilian program, the implementation, use, and outcomes of the AGHU are patchy among university hospitals.

The Brazilian program experienced the same difficulties experienced by other countries implementing HIS on a large scale [7]. Furthermore, the Brazilian implementers needed to manage the program in a country of immense geographic proportion and with a wide diversity of cultural and territorial characteristics. This is how the director of a HU expresses himself: “*You can imagine, doing something in England—a small country, a small population—is already quite complex; now you can imagine me doing it in 40 hospitals, in different regions, with different realities and each of them with a different culture*” (G8).

Essentially, the need to align the differences among the 40 hospitals that are part of the EBSERH network is influenced by the diversity of the Brazilian environment. Difficulties of compliance and differences in local and regional context exist when looking for a standardized solution which, in turn, leads to the emergence of barriers in the implementation and adoption of the AGHU. In addition, the political and economic changes in the national scenario over time, and consequently in the program’s governance structure, contributed to resistance and frustration of users’ expectations: clearly an important influence when considering that large-scale implementation of HIS is shaped by their governance structure [42,45,46].

Conflicts among stakeholders [43,47] resulting from EBSERH centralized management; the top-down governance [26], limiting hospitals’ and local IT teams’ autonomy [48]; the sizing of the IT team for the development of new modules and updates; the segmented availability of system modules and the slow system development, which occurred parallel to the implementation, generated considerable delays. These aspects contributed to the differences in the number of modules implemented in each HU and the outcomes obtained by each of them over time. Furthermore, the number of HUs in the EBSERH network grew over time, given the prerogative of voluntary adherence to centralized management, often not allowing comprehensive planning and increasing the scope and complexity of program management. Underestimation of program size and level of complexity was also identified as a barrier in several countries [12,49].

Aspects at the macro level created barriers to the national implementation program of AGHU with subsequent implications, many of which were not dimensioned by government agencies, in the implementation at the local level (meso). For example, local planning actions were impacted by the absence or lack of communication of comprehensive national planning, aimed at making available and introducing modules and updated versions. Functional gaps in AGHU modules, as well as the failure to “deliver” a complete solution, demand the development of control systems and mechanisms in hospitals, to be used concurrently with AGHU. The concomitant use of these local systems makes IT management difficult due to the development of methods for integrating parallel systems with the main system (AGHU).

It must be considered that the success of a large-scale IT implementation program does not depend only on actions at the national level, but on a set of local aspects linked to each HU [7,28]. The intervening aspects of the national program related to the organizational context of each hospital (meso level) and the different forms that are configured in each HU thus facilitating and/or frustrating the roll-out of the national program. For example, in some hospitals, the strong commitment and involvement of senior management; change management actions; the effective performance of interdisciplinary committees for the local management of the project; the different training strategies; and initiatives to exchange experiences with more advanced HUs in the process, contributed to the advancement of implementation in that context. On the other hand, the evidence of limitations regarding these aspects in other hospitals caused delays and maximized local resistance to the adoption of AGHU.

Resistance to the use of the system from professional groups (micro) affected aspects related to the hospital context (meso). This resistance comes, in part, from skepticism about the benefits obtained with the use of technology and/or inexperience with its use; from dissatisfaction with changes in their practices, and from comparisons to other known systems. It is up to local management to provide specific actions to minimize the resistance of professionals, influencing the way the implementation process is managed locally. Furthermore, it was identified that the national manager uses the experience of each HU in the system implementation, the strategies used, and the lessons learned locally (meso) for the improvement of the AGHU and benchmarking in the network in a broader sense (macro).

The varied interactions among the intervening aspects at different levels of the analysis affected the national program in several ways. Although all the benefits resulting from the program cannot yet be perceived equally in all HUs in the network, the outcomes perceived by managers and users of the HUs in the more advanced stages of the implementation process have contributed to improvements. Namely, these are in the quality and safety of care practice, in aspects related to management support, in addition to increasing hospital processes and operational efficiency. These contributions encompass the national, institutional, and individual levels.

From a perspective of IT’s contribution to development [15,50], economic benefits come from the improvements achieved in operational efficiency and resource management, resulting in the reduction of administrative costs and the possibility of greater investment in strategic areas. The implementation of AGHU has contributed to decision-making by health managers based on concrete information, improving strategic planning and resource allocation in each hospital and globally in the hospital network.

In addition, some contributions in the social and human dimensions can also be observed. More efficient coordination and integration of patient care have been achieved, reducing duplication of procedures, prescription and evolution errors, as well as patient waiting time. These benefits have increased the quality and safety of care. Students and professors in the health area also find advantages, as they can have easy access to information related to real clinical cases, enriching education and scientific research.

The increased integration between hospitals and the healthcare network has led to closer collaborations among professionals from different hospitals, disseminating knowledge and best practices nationwide. This collaborative approach can contribute to improving access to healthcare and reducing regional disparities, thereby enhancing the quality of life for various communities served by university hospitals.

Despite the evident contributions, this study reinforces that the nationwide implementation of a hospital information system is not without challenges. Intervening factors in the process can hinder the full adoption of technology and even substantially increase the financial resources involved in the implementation.

Although IT can bring numerous benefits to development in the healthcare domain, in national implementations, barriers at different levels (macro, meso, and micro), amplified in emerging economies and in more complex contexts, such as university hospitals, can limit such benefits. Effective IT action depends on seeing it as part of a broader set of approaches to address health issues [13,51].

We defend that this vision needs to be shared and taken as a basis for the actions not only of national managers but essentially, of each of the institutions (hospitals) and professionals involved. This research identified that in hospitals where actions were developed to strengthen the software-people-institutions nexus [13], system outcomes for health domains were achieved faster and more effectively. This process was fed back when the hospitals saw actions by the national manager (at the macro level) that also strengthened this nexus. In this sense, we understand that in nationwide health IT implementations, the nexus now expanded to software-people-institutions-network, is crucial for the development of the institution’s network as a whole, the hospital institutions, the professionals and health students involved, and consequently for the generation of more effective health outcomes for patients.

## 7. Conclusions

The national program for the implementation of an integrated information system in the country’s university hospitals was affected by several aspects related to the broader context of system implementation; the structural, cultural, and political context of each of the hospitals involved; and referring to the individual, technology and work activities. These aspects acted as barriers and/or facilitators, and the dynamic and complex interactions established among them influenced the implementation process, including the long duration of the national program and the unequal outcomes between the HUs in the national network. The research findings show dynamic interactions at the intra and inter-level of analysis (macro, meso, and micro) that shaped the implementation process and its outcomes over time. The intervening aspects identified are configured in different ways in each hospital, influencing the system implementation process. This fact reinforces the diversity of the context of each institution, the lack of homogeneity in the actions to introduce AGHU in the different HUs, as well as the barriers related to the simultaneous development and implementation of the system.

The evidence related to the main outcomes of the AGHU implementation process demonstrates a clear evolution in hospitals’ performance (especially those that are at a more advanced stage) and contributes to the health care of the population, teaching, and research. Progress related to management, as well as the productivity of resources and the improvement in the quality of the care process at these hospitals, justifies the investment in improving the tool, expanding the scope of functionality, and optimizing the implementation process.

However, the identified limiting aspects of the AGHU implementation have contributed to delays in this process that have taken already more than a decade. These issues and their approaches require understanding and attention from all hospital managers involved in the large-scale implementation of HIS government programs in order to maximize the benefits they can offer. The difficulties faced by the HUs and by the national managers demonstrate the complexity of the implementation of a health management system nationally in a developing country. This is amplified by the specifics of the regional, local and hospital context as well as by the very nature of a university hospital itself.

The ability to consider the different institutional and social contexts in which the technology will be introduced and to manage these differences is highly relevant to achieving the full benefit from these governmental programs. Technology alone cannot reach its full transformative potential. It is important to recognize the challenges faced in the process and emphasize the relevance of an integrative and cooperative approach to overcome them and contribute to the different perspectives of development.

The research presented here has implications for the literature on the large-scale implementation and evaluation of IT in the health sector and on the management of complex programs and projects. Based on the experiences involved in implementing an innovative government program in the network of university hospitals in a developing country, this investigation highlights the facilitating and limiting aspects of the implementation process, their interrelationships as well and their outcomes. Additionally, the structure of intervening aspects and outcomes arising from the findings of this research can be used in future studies focused on analyzing and evaluating the implementation and adoption of HIS technology on a large-scale.

The results of the study for practice may help decision-makers understand the complexity involved in the implementation process of large-scale systems in university hospitals and help with strategic decisions on how to improve the chances of success. National and local managers should collaborate in order to develop strategies to minimize the limiting aspects and enhance the facilitating aspects identified in this study, by considering the importance of the involvement of all levels and the context of each hospital. In addition, the Brazilian experience of integrating HUs and the results that have been achieved with the AGHU implementation program nationally can provide insights for other countries.

The limitations of this research lie in the number of participating hospitals, 21 out of a total of 40 HUs that make up the EBSERH network. Thus, the results may not be fully representative of all HUs in the national network. A way to increase the participation of a greater number of hospitals could be through the support of the national manager of the EBSERH hospital network and by disseminating the results of this research to the hospital managers.

Considering that new AGHU modules continue to be created and the system continues to grow, collecting data over a time window may limit the findings of the program’s evolution. Therefore, it would be useful to replicate and expand the scope of the research to include longitudinal studies of health IT implementation programs to understand the evolution of the aspects identified here over time. Additionally, the use of the structure of intervening aspects and outcomes of the implementation of this study is suggested to verify its adherence to other contexts and to improve it. Research focused on a more in-depth analysis of relationships between aspects of the macro, meso, and micro levels identified in this investigation can also contribute to the advancement of knowledge in the field.

## Figures and Tables

**Table 1:** Units of analysis 1 to 5 [10].

	Analysis Unit 1 University Hospital 1 (HU1)	Analysis Unit 2 University Hospital 2 (HU2)	Analysis Unit 3 University Hospital 3 (HU3)	Analysis Unit 4 University Hospital 3 (HU4)	Analysis Unit 5 University Hospital 5 (HU5)
Region	Northern	Midwest	Southeast	Southern	Northeast
Year Established	1957	1984	1982	1959	2008
Number of beds	221	124	384	403	139
Total number of employees	1741	809	2059	2376	725
Attendances (annual average)	64.662	141.660	926.329	382.362	80.868

**Table 2:** Unit of analysis 6 [10].

Analysis Unit 6 Network of HUs (21 University Hospitals Survey Participants)	
Region	Number of HUs
Northern	3
Midwest	4
Southeast	5
Southern	4
Northeast	5
The average number of beds	180
Average number of employees	1.438
Average number of attendances (annual)	384.479

**Table 3:** Interview script.

Topic	Description
1	Characterization of the participants
2	Scenario prior to the adoption of the AGHU
3	Implementation process: intervening aspects in the implementation process considering the macro, meso, and micro levels of analysis and the various stages of the implementation (Figure 1)
4	Perceived outcomes of the implementation process
5	Comments and suggestions for the government program manager (EBSERH) and for HUs less advanced in the implementation process

**Table 4:** Questionnaire sections and aspects.

Questionnaire Sections	Aspects	
1 Characterization of respondents	Respondent’s hospital, Area of expertise, Length of service at HU, Member of the implementation team, Engagement in management activities, Duration of AGHU usage	
2 National AGHU program	Macro	Government IT policy; National governance framework; HU’s involvement; Alignment and communication among stakeholders; Government commitment; Financial investment; Government evaluation.
3 Planning, implementation, and use of the AGHU	Meso	Awareness campaigns; Infrastructure and equipment; Participation in development and implementation; Explicit leadership; Human resources; Intraorganizational communication; Timetables; Management commitment; Engagement and resistance actions; Training and technical support; HU’s hierarchical structure and organizational culture; AGHU evaluation.
	Micro	User motivation; Computer skills; Technical and operational aspects of the system; Available features; AGHU user-friendliness; Process complexity; Alignment of servers/processes; User satisfaction with AGHU.
4 Outcomes	Service quality; Job quality; Work practices; Productivity; Management processes.	

**Table 5:** Emerging themes and sub-themes: intervening aspects at the macro level (F—facilitators aspects, L—limiting aspects).

1—National political and economic scenario	Presidential changes and political and economic instabilities	L
	Alternation of managers of the network of HUs	L
2—Territorial dimensions and regional/local differences	Differences in hospital geographic location, size and structure	L
	Differences in processes, language and maturity of previous systems	L
3—National health and e-health policies	Unification of HUs in a single network and centralized management (EBSERH)	F
	Voluntary initial adherence to the unification policy	L
	Single IT policy and standard hospital management system (AGHU)	F/L
	Medium and long-term IT policy planning	L
	Lack of interoperability standards between systems and a single e-health policy for the Unified Health System (SUS)	L
4—Financing	Financing of human and material resources to revitalize the HUs	F
	IT budget availability	F
	Resource limitation and competition between hospital and IT equipments	L
5—Government support and involvement	Top management commitment to the process	F/L
	Valuing and prioritizing the implementation and sustainability of the AGHU	F
6—Governance of the implementation process	Mandatory adoption	F/L
	Centralization of system development and technical support	F/L
	Communication process among governance and the HUs	F/L
	Promoting experience and knowledge sharing among network HUs	F/L
	HUs continuous training program	F/L
	Dimensioning of the IT team and knowledge of care processes	L
	Continuous performance evaluation program	L
7—System design and implementation plan	Concurrent system design, development and implementation	L
	Delays in system development, implementation and updates	L
	Implementation process planning	L
	System, implementation and lessons learned documentation	L
	Participation of hospitals in the implementation of the system	F
	Conducting beta tests and pilot tests	F
	Lack of contingency plan	L
8—Relationships between stakeholders	Conflicts of interest between different groups regarding national policies	L
	Conflicting views regarding the adoption of a standard system for HUs	L

**Table 6:** Emerging themes and sub-themes: intervening aspects at the meso level (F—facilitators aspects, L—limiting aspects).

1—Management commitment and support	Commitment of managers to the implementation process	F/L
	Alignment among managers	F/L
2—Organizational culture	Changes in institutionalized practices	L
	Culture receptive to change	F
3—Organizational structure	Standardization of local organizational structures	F
	IT area directly linked to local top management and local IT sectors	F
	Definition of workflows and work processes	L
4—People Management	Definition of competencies and user profiles	L
	Differences in the hiring system	L
	Staff turnover, work overload and reduced staff	L
5—Change management	Support from residents to groups with little familiarity with technology	F
	Communication and awareness about the implementation process	F/L
	Strategies for integrating new employees and residents	F/L
	Prioritization and institutionalization of system use	F
	Key users on local governance and implementation teams	F
	Training programs to increase engagement	F
6—Local governance of the implementation process	Multidisciplinary AGHU management committee	F/L
	Local communication and user participation	F/L
	Experiences sharing between advanced and beginner HUs	F
7—IT resources and capabilities	Features of previous systems	F/L
	IT infrastructure for system use (previous and current)	F/L
	Local IT staff sizing and system knowledge	L
	Availability and agility of local technical support	F/L
	Dependency on system and network availability	L
8—Training	Initial and continued training	F/L
	Definition of multipliers	F
	Different training modalities and strategies	F
9—Evaluation	Local monitoring of implementation and usage	F
	Master plan and indicators for local evaluation	F

**Table 7:** Emerging themes and sub-themes: intervening aspects at the micro level (F—facilitators aspects, L—limiting aspects).

1—Technology	Alignment of functionalities with hospital practices	F/L
	Features not covered by the system	L
	System maturity	L
	Usability	F/L
	Technical aspects	L
2—Individuals	Skepticism, unrealistic expectations, frustration of expectations by slowing system development	L
	Expectation for qualification of management and process improvement	F
	Previous experiences with integrated hospital information systems	F/L
	Positive or negative experiences using the system	F/L
	Resistances	L
	Ability to use technology (age or length of career)	F/L
	Personal interest and motivation	F/L
	Inappropriate or incorrect use of the system	L
3—Health activities and processes	High complexity of work processes and activities	L
	Research, teaching, and learning linked to hospital processes	L

**Table 8:** Themes and sub-themes emerging from the outcomes of AGHU implementation.

*Category*	*Subcategories/Results*	*Illustrative Excerpts*
1—Work practices and processes	Organization, standardization and improvement of organizational processes.	*“We changed and improved many processes” (U4) “[…] it facilitated the management of hospital practice” (G11).*
	Incorporation of new management practices and work procedures.	
2—Planning, control and decision making	Productivity and inputs monitoring, and planning support.	*“Greater control of laboratory tests, patient and students service” (G6); “You have information to make a decision” (G10).*
	Integrated management and decision-making support.	
3—Transparency/accountability	Generation of local indicators of assistance production.	*“Extraction of indicators and you can do a series of analyzes […] through these indicators, the headquarters can compare the hospitals” (G14).*
	Generation of national indicators of assistance production.	
4—Optimization in the use of resources	Optimization of the use of care resources (staff, rooms, beds, inputs)	*“Increase in the number of consultations” (G5); “coordinated management of the outpatient clinic, avoiding loss of time, reducing operating costs, reducing paper consumption”. (G1);*
	Rationalization of the use of inputs by reducing the use of paper.	
5—Professional productivity	Agility of assistance and administrative processes	*“Greater agility in dispensing and carrying out exams. “(G9); “The process has become much more agile.” (G13); “It has improved productivity and service time as well. (G6).*
	Increased care productivity	
6—Security of patient information	Confidentiality of patient data	*“A framework of reserves, secrecy, confidentiality, property, in short, that needs to be protected.” (G3); “The patient can visualize his entire history (G24).*
	Preservation of patient information	
7—Safety and quality of care	Integration and ease of access to information relevant to care (schedule, evolution, registration of practices and exams)	*“It makes it easier for all teams to follow up on patient care.” (G5); “we surpassed the doctor’s handwriting, because you transcribed one thing and there at the pharmacy, they read another” (G1); “The AGHU ties the prescription to a series of factors, and before there was total liberality, I think this generated a large margin of error.” (G18); “A violation there of what is established within the records, within the professional behavior of medical violation, the AGHU shows you more easily […] it allows you to monitor professional practice” (G3).*
	Better use of service time	
	Prescription error reduction	
	Reduction in patient evolution errors by establishing protocols and standardizing care practices.	
	Reduced patient waiting time	
	Safety in the care process for professionals and patients	
	Transparency of care practice for professionals and patients	
8—Improvement in teaching and research	Knowledge and learning generated by interaction with the system.	*“The resident learns a lot […] a good part of the residents help the staff to evolve at AGHU (G5); “the generation of knowledge, the extraction of information and the crossing of these in the use of research” (G14); “This, for research, is a full plate, as you no longer need to fiddle with paper” (G22).*
	Ease of access to care information for clinical case studies, scientific work and research.	

## Data Availability

Data is unavailable due to privacy and ethical restrictions.
